# Finding shortest lattice vectors faster using quantum search

**DOI:** 10.1007/s10623-015-0067-5

**Published:** 2015-04-14

**Authors:** Thijs Laarhoven, Michele Mosca, Joop van de Pol

**Affiliations:** 1 Eindhoven University of Technology, Eindhoven, The Netherlands; 2 Institute for Quantum Computing and Department of Combinatorics & Optimization, University of Waterloo, Waterloo, ON Canada; 3 Perimeter Institute for Theoretical Physics, Waterloo, ON Canada; 4 Canadian Institute for Advanced Research, Toronto, Canada; 5 University of Bristol, Bristol, UK

**Keywords:** Lattices, Shortest vector problem, Sieving, Quantum search, 52C07, 68W01, 81P68, 94A60

## Abstract

By applying a quantum search algorithm to various heuristic and provable sieve algorithms from the literature, we obtain improved asymptotic quantum results for solving the shortest vector problem on lattices. With quantum computers we can provably find a shortest vector in time $2^{1.799n + o(n)}$, improving upon the classical time complexities of $2^{2.465n + o(n)}$ of Pujol and Stehlé and the $2^{2n + o(n)}$ of Micciancio and Voulgaris, while heuristically we expect to find a shortest vector in time $2^{0.268n + o(n)}$, improving upon the classical time complexity of $2^{0.298n + o(n)}$ of Laarhoven and De Weger. These quantum complexities will be an important guide for the selection of parameters for post-quantum cryptosystems based on the hardness of the shortest vector problem.

## Introduction

Large-scale quantum computers will redefine the landscape of computationally secure cryptography, including breaking public-key cryptography based on integer factorization or the discrete logarithm problem [75] or the principle ideal problem in real quadratic number fields [37], providing sub-exponential attacks for some systems based on elliptic curve isogenies [23], speeding up exhaustive searching [15, 35], counting [18] and (with appropriate assumptions about the computing architecture) finding collisions and claws [4, 17, 19], among many other quantum algorithmic speed-ups [22, 61, 76].

Currently, a small set of systems [12] are being studied intensely as possible systems to replace those broken by large-scale quantum computers. These systems can be implemented with conventional technologies and to date seem resistant to substantial quantum attacks. It is critical that these systems receive intense scrutiny for possible quantum or classical attacks. This will boost confidence in the resistance of these systems to (quantum) attacks, and allow us to fine-tune secure choices of parameters in practical implementations of these systems.

One such set of systems bases its security on the computational hardness of certain lattice problems. Since the late 1990s, there has been a lot of research into the area of lattice-based cryptography, resulting in encryption schemes [39, 60, 68], digital signature schemes [26, 33, 56] and even fully homomorphic encryption schemes [16, 32]. Each of the lattice problems that underpin the security of these systems can be reduced to the shortest vector problem [77]. Conversely, the decisional variant of the shortest vector problem can be reduced to the average case of such lattice problems. For a more detailed summary on the security of lattice-based cryptography, see [49, 77].

In this paper, we closely study the best-known algorithms for solving the shortest vector problem, and how quantum algorithms may speed up these algorithms. By challenging and improving the best asymptotic complexities of these algorithms, we increase the confidence in the security of lattice-based schemes. Understanding these algorithms is critical when selecting key-sizes and other security parameters. Any non-trivial algorithmic advance has the potential to compromise the security of a deployed cryptosystem, for example in [13] an improvement in the index calculus method for finding discrete logarithms led to the break of a Diffie–Hellman system that had been deployed in software and was in the process of being implemented in hardware.

### Lattices

Lattices are discrete subgroups of $\mathbb {R}^n$. Given a set of $n$ linearly independent vectors $B = \{\mathbf {b}_1, \ldots , \mathbf {b}_n\}$ in $\mathbb {R}^n$, we define the lattice generated by these vectors as $\fancyscript{L} = \left\{ \sum _{i = 1}^n \lambda _i \mathbf {b}_i: \lambda _i \in \mathbb {Z}\right\}$. We call the set $B$ a basis of the lattice $\fancyscript{L}$. This basis is not unique; applying a unimodular matrix transformation to the vectors of $B$ leads to a new basis $B'$ of the same lattice $\fancyscript{L}$.

In lattices, we generally work with the Euclidean or $\ell _2$-norm, which we will denote by $\Vert \cdot \Vert$. For bases $B$, we write $\Vert B\Vert = \max _i \Vert \mathbf {b}_i\Vert$. We refer to a vector $\mathbf {s} \in \fancyscript{L} \setminus \{\mathbf {0}\}$ such that $\Vert \mathbf {s}\Vert \le \Vert \mathbf {v}\Vert$ for any $\mathbf {v} \in \fancyscript{L} \setminus \{\mathbf {0}\}$ as a shortest (non-zero) vector of the lattice. Its length is denoted by $\lambda _1(\fancyscript{L})$. Given a basis $B$, we write $\fancyscript{P}(B) = \left\{ \sum _{i = 1}^n \lambda _i \mathbf {b}_i: 0 \le \lambda _i < 1\right\}$ for the fundamental domain of $B$.

One of the most important hard problems in the theory of lattices is the *shortest vector problem* (SVP). Given a basis of a lattice, the shortest vector problem consists of finding a shortest non-zero vector in this lattice. In many applications, finding a reasonably short vector instead of a shortest vector is also sufficient. The *approximate shortest vector problem* with approximation factor $\delta$ (SVP$_{\delta }$) asks to find a non-zero lattice vector $\mathbf {v} \in \fancyscript{L}$ with length bounded from above by $\Vert \mathbf {v}\Vert \le \delta \cdot \lambda _1(\fancyscript{L})$.

Finding short vectors in a lattice has been studied for many reasons, including the construction of elliptic curve cryptosystems [7, 28, 29], the breaking of knapsack cryptosystems [25, 51, 57, 66] and low-exponent RSA [24, 78], and proving hardness results in Diffie–Hellman-type schemes [14]. For appropriately chosen lattices, the shortest vector problem appears to be hard, and may form the basis of new public-key cryptosystems.

### Finding short vectors

The approximate shortest vector problem is integral in the cryptanalysis of lattice-based cryptography [30]. For small values of $\delta$, this problem is known to be NP-hard [2, 44], while for certain exponentially large $\delta$ polynomial time algorithms are known to exist that solve this problem, such as the celebrated LLL algorithm of Lenstra et al. [52, 57]. Other algorithms trade extra running time for a better $\delta$, such as LLL with deep insertions [73] and the BKZ algorithm of Schnorr and Euchner [73].

The current state-of-the-art for classically finding short vectors is BKZ 2.0 [21, 72], which is essentially the original BKZ algorithm with the improved SVP subroutine of Gama et al. [31]. Implementations of this algorithm, due to Chen and Nguyen [21] and Aono and Naganuma [8], currently dominate the SVP and lattice challenge hall of fame [53, 71] together with a yet undocumented modification of the random sampling reduction (RSR) algorithm of Schnorr [74], due to Kashiwabara et al. [71].

In 2003, Ludwig [55] used quantum algorithms to speed up the original RSR algorithm. By replacing a random sampling from a big list by a quantum search, Ludwig achieves a quantum algorithm that is asymptotically faster than its classical counterpart. Ludwig also details the effect that this faster quantum algorithm would have had on the practical security of the lattice-based encryption scheme NTRU [39], had there been a quantum computer in 2005.

### Finding shortest vectors

Although it is commonly sufficient to find a short vector (rather than a *shortest* vector), the BKZ algorithm and its variants all require a low-dimensional exact SVP solver as a subroutine. In theory, any of the known methods for finding a shortest vector could be used. We briefly discuss the three main classes of algorithms for finding shortest vectors below.

*Enumeration* The classical method for finding shortest vectors is enumeration, dating back to work by Pohst [64], Kannan [43] and Fincke and Pohst [27] in the first half of the 1980s. In order to find a shortest vector, one enumerates all lattice vectors inside a giant ball around the origin. If the input basis is only LLL-reduced, enumeration runs in $2^{O(n^2)}$ time, where $n$ is the lattice dimension. The algorithm by Kannan uses a stronger preprocessing of the input basis, and runs in $2^{O(n \log n)}$ time. Both approaches use only polynomial space in $n$.

*Sieving* In 2001, Ajtai et al. [3] introduced a technique called sieving, leading to the first probabilistic algorithm to solve SVP in time $2^{O(n)}$. Several different sieving methods exist, but they all rely on somehow saturating the space of short lattice vectors, by storing all these vectors in a long list. This list will inevitably be exponential in the dimension $n$, but it can be shown that these algorithms also run in single exponential time, rather than superexponential (as is the case for enumeration). Recent work has also shown that the time and space complexities of sieving improve when working with ideal lattices [40, 70], leading to the current highest record in the ideal lattice challenge hall of fame [63].

*Computing the Voronoi cell* In 2010, Micciancio and Voulgaris presented a deterministic algorithm for solving SVP based on constructing the Voronoi cell of the lattice [58]. In time $2^{2n + o(n)}$, this algorithm is able to construct an exact $2^{n + o(n)}$-space description of the Voronoi cell of the lattice, which can then be used to solve both SVP and CVP. The overall time complexity of $2^{2n + o(n)}$ was until late 2014 the best known complexity for solving SVP in high dimensions.^1^

*Discrete Gaussian sampling* Very recently, an even newer technique was introduced by Aggarwal et al. [1], making extensive use of discrete Gaussians on lattices. By initially sampling $2^{n + o(n)}$ lattice vectors from a very wide discrete Gaussian distribution (with a large standard deviation), and then iteratively combining and averaging samples to generate samples from a more narrow discrete Gaussian distribution on the lattice, the standard deviation can be reduced until the point where a set of many samples of the resulting distribution is likely to contain a shortest non-zero vector of the lattice. This algorithm runs in provable $2^{n + o(n)}$ time and space.

*Practice* While sieving, the Voronoi cell algorithm, and the discrete Gaussian sampling algorithm have all surpassed enumeration in terms of classical asymptotic time complexities, in practice enumeration still dominates the field. The version of enumeration that is currently used in practice is due to Schnorr and Euchner [73] with improvements by Gama et al. [31]. It does not incorporate the stronger version of preprocessing of Kannan [43] and hence has an asymptotic time complexity of $2^{O(n^2)}$. However, due to the larger hidden constants in the exponents and the exponential space complexity of the other algorithms, enumeration is actually faster than other methods for most practical values of $n$. That said, these other methods are still relatively new and unexplored, so a further study of these other methods may tip the balance.

### Quantum search

In this paper we will study how quantum algorithms can be used to speed up the SVP algorithms outlined above. More precisely, we will consider the impact of using Grover’s quantum search algorithm [35], which considers the following problem.

Given a list $L$ of length $N$ and a function $f: L \rightarrow \{0, 1\}$, such that the number of elements $e \in L$ with $f(e) = 1$ is small. Construct an algorithm “${{\mathrm{{\text {Search}}}}}$” that, given $L$ and $f$ as input, returns an $e \in L$ with $f(e) = 1$, or determines that (with high probability) no such $e$ exists. We assume for simplicity that $f$ can be evaluated in unit time.

*Classical algorithm* With classical computers, the natural way to find such an element is to go through the whole list, until one of these elements is found. This takes on average $O(N)$ time. This is also optimal up to a constant factor; no classical algorithm can find such an element in less than $\varOmega (N)$ time.

*Quantum algorithm* Using Grover’s quantum search algorithm [15, 18, 35], we can find such an element in time $O(\sqrt{N})$. This is optimal up to a constant factor, as any quantum algorithm needs at least $\varOmega (\sqrt{N})$ evaluations of $f$ [10].

Throughout the paper, we will write $x \leftarrow {{\mathrm{{\text {Search}}}}}\{e \in L: f(e) = 1\}$ to highlight subroutines that perform a search in some long list $L$, looking for an element $e \in L$ satisfying $f(e) = 1$. This assignment returns true if an element $e \in L$ with $f(e) = 1$ is found (and assigns such an element to $x$), and returns false if no such $e$ exists. This allows us to give one description for both the classical and quantum versions of each algorithm, as the only difference between the two versions is which version of the subroutine is used.

### RAM model

For both the classical and the quantum versions of these search algorithms, we assume a RAM model of computation where the $j$th entry of the list $L$ can be looked up in constant time (or polylogarithmic time). In the case that $L$ is a virtual list where the $j$th element can be computed in time polynomial in the length of $j$ (thus polylogarithmic in the length of the list $L$), then look-up time is not an issue. When $L$ is indeed an unstructured list of values, for classical computation, the assumption of a RAM-like model has usually been valid in practice. However, there are fundamental reasons for questioning it [11], and there are practical computing architectures where the assumption does not apply. In the case of quantum computation, a practical RAM-like quantum memory (e.g. [34]) looks particularly challenging, especially for first generation quantum computers. Some authors have studied the limitations of quantum algorithms in this context [11, 36, 41].

Some algorithms (e.g. [4]) must store a large database of information in regular quantum memory (that is, memory capable of storing quantum superpositions of states). In contrast, quantum searching an actual list of $N$ (classical) strings requires the $N$ values to be stored in quantumly addressable classical memory (e.g. as Kuperberg discusses in [45, 46]) and $O(\log N)$ regular qubits. Quantumly addressable classical memory in principle could be much easier to realize in practice than regular qubits. Furthermore, quantum searching for a value $x \in \{0,1\}^n$ satisfying $f(x) =1$ for a function $f: \{0,1\}^n \rightarrow \{0,1\}$ which can be implemented by a circuit on $O(n)$ qubits only requires $O(n)$ regular qubits, and there is no actual list to be stored in memory. In this paper, the quantum search algorithms used require the lists of size $N$ to be stored in quantumly addressable classical memory and use $O(\log N)$ regular qubits and $O(\sqrt{N})$ queries into the list of numbers.

In this work, we consider (conventional) classical RAM memories for the classical algorithms, and RAM-like quantumly addressable classical memories for the quantum search algorithms. This is both a first step for future studies in assessing the impact of more practical quantum architectures, and also represents a more conservative approach in determining parameter choices for lattice-based cryptography that should be resistant against the potential power of quantum algorithmic attacks. Future work may also find ways to take advantage of advanced quantum search techniques, such as those surveyed in [69].

### Contributions

In this paper, we show that quantum algorithms can significantly speed up various sieving algorithms from the literature. The constants in the time exponents generally decrease by approximately $25\, \%$, leading to an improvement in both the best provable (exact and approximate) and the best heuristic asymptotic results for solving the shortest vector problem:

- Provably, we can find a shortest vector in any lattice in time $2^{1.799n + o(n)}$. (Without quantum search, the best provable algorithm (see footnote 1) runs in time $2^{2.000n + o(n)}$.)
- Heuristically, we can find a shortest vector in any lattice in time $2^{0.286n + o(n)}$. (Without quantum search, the best heuristic algorithm runs in time $2^{0.337n + o(n)}$.)
- Provably, we can solve SVP$_{\delta }$ in any lattice in time $2^{0.603n + o_{\delta }(n)}$.^2^ (Without quantum search, the best provable algorithm runs in time $2^{0.804n + o_{\delta }(n)}$.)

Table 1 contains an overview of classical and quantum complexities of various SVP algorithms, and summarizes the results in this paper. While the Voronoi cell algorithm [58] is asymptotically the best algorithm in the provable classical setting (see footnote 1), we show that with quantum search, both the AKS-Birthday algorithm described by Hanrot et al. [38] and the ListSieve-Birthday algorithm of Pujol and Stehlé [65] surpass the $2^{2n + o(n)}$ time complexity of the Voronoi cell algorithm. While the main focus in this paper is on sieving algorithms, we also briefly consider applying quantum search to other methods, and argue why applying the same techniques does not easily lead to significant speed-ups for those algorithms.

After the initial submission of our paper, it was shown that the provable time complexity of solving SVP can be further improved to $2^{n + o(n)}$ using a new method based on discrete Gaussian sampling [1]. Since the provable time complexity of sieving (using quantum search) is asymptotically higher than $2^{n + o(n)}$, this means that sieving on a quantum computer is no longer the best provable algorithm (asymptotically) for solving SVP exactly. In Sect. 9.3 we therefore also discuss the impact that quantum search may have on the discrete Gaussian sampling method.

**Fig. 1:**
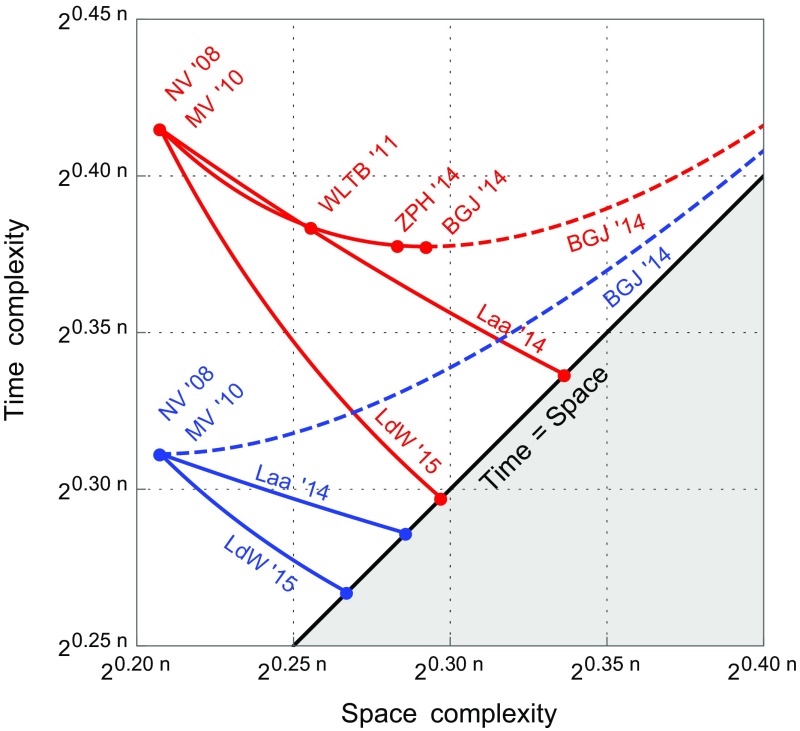
The heuristic space-time trade-off of various heuristic sieve algorithms from the literature (*red*), and the heuristic trade-offs obtained with quantum search applied to these algorithms (*blue*). The optimized 2-Level-Sieve and 3-Level-Sieve of Wang et al. and Zhang et al. both collapse to the point $(2^{0.208n}, 2^{0.312n})$ as well. *Dashed lines* are increasing in both the time and the space complexity, and therefore do not offer a useful trade-off (Color figure online)

The heuristic improvements obtained with quantum search are also shown in Fig. 1. This figure also shows the tunable trade-offs that may be obtained with various classical and quantum sieving algorithms (rather than just the single entries given in Table 1). As can be seen in the figure, we only obtain a useful trade-off between the quantum time and space complexities for the HashSieve and SphereSieve algorithms; for other algorithms the trade-offs are not really trade-offs, as both the time and the space complexity increase by changing the parameters.

**Table 1:** A comparison of time and space complexities of SVP algorithms, both classically and quantumly

Algorithm	Classical		Quantum		Roadmap
Name [references]	$\log _2 (\hbox {time})$	$\log _2 (\hbox {space})$	$\log _2 (\hbox {time})$	$\log _2 (\hbox {space})$	
Provable SVP					
*Enumeration* *algorithms*	$\varOmega (n \log n)$	$O(\log n)$	$\varOmega (n \log n)$	$O(\log n)$	(Sect. 9.1)
AKS-Sieve [3, 38, 59, 62, 67]	$3.398n$	$1.985n$	$2.672n$	$1.877n$	(Sect. 8.1)
ListSieve [59]	$3.199n$	$1.327n$	$2.527n$	$1.351n$	(Sect. 8.2)
AKS-Sieve- Birthday [38]	$2.648n$	$1.324n$	$1.986n$	$1.324n$	(Sect. 8.3)
ListSieve- Birthday [65]	$2.465n$	$1.233n$	$1.799n$	$1.286n$	(Sect. 2)
*Voronoi cell* *algorithm*	$2.000n$	$1.000n$	$2.000n$	$1.000n$	(Sect. 9.2)
*Discrete Gaussian* *sampling*	$\mathbf {1.000n}$	$0.500n$	$\mathbf {1.000n}$	$0.500n$	(Sect. 9.3)
Heuristic SVP					
NV-Sieve [62]	$0.415n$	$0.208n$	$0.312n$	$0.208n$	(Sect. 3)
GaussSieve [59]	$0.415n$	$0.208n$	$0.312n$	$0.208n$	(Sect. 4)
2-Level-Sieve [79]	$0.384n$	$0.256n$	$0.312n$	$0.208n$	(Sect. 8.4)
3-Level-Sieve [80]	$0.378n$	$0.283n$	$0.312n$	$0.208n$	(Sect. 8.5)
Overlattice- Sieve [9]	$0.378n$	$0.293n$	$0.312n$	$0.208n$	(Sect. 8.6)
HashSieve [47]	$0.337n$	$0.337n$	$0.286n$	$0.286n$	(Sect. 5)
SphereSieve [48]	$\mathbf {0.298n}$	$0.298n$	$\mathbf {0.268n}$	$0.268n$	(Sect. 6)
Prov. SVP$_{\delta }$					
*Enumeration* *algorithms*	$\varOmega (n \log n)$	$O(\log n)$	$\varOmega (n \log n)$	$O(\log n)$	(Sect. 9.1)
*Voronoi cell* *algorithm*	$2.000n$	$1.000n$	$2.000n$	$1.000n$	(Sect. 9.2)
*Discrete Gaussian* *sampling*	$1.000n$	$0.500n$	$1.000n$	$0.500n$	(Sect. 9.3)
ListSieve- Birthday [54]	$\mathbf {0.802n}$	$0.401n$	$\mathbf {0.602n}$	$0.401n$	(Sect. 7)

Except for the italicized algorithms, these are all results based on sieving. The top rows describe provable algorithms for SVP, the middle rows describe heuristic algorithms for SVP, and the bottom rows describe provable algorithms for solving SVP$_{\delta }$, and their asymptotic complexities as $\delta , n \rightarrow \infty$

Entries in bold indicate the best known asymptotic time complexities to date

### Outline

The outline of this paper is as follows, and can also be found in Table 1. In Sect. 2 we first consider the current best provable sieving algorithm for solving the shortest vector problem, the ListSieve-Birthday algorithm of Pujol and Stehlé [65]. This is the birthday paradox variant of the ListSieve algorithm of Micciancio and Voulgaris [59] (which is briefly described in Sect. 8.2), and we get the best provable quantum time complexity by applying quantum search to this algorithm. In Sects. 3 and 4 we then consider two of the most important heuristic sieving algorithms to date, the NV-Sieve algorithm of Nguyen and Vidick [62] and the GaussSieve algorithm of Micciancio and Voulgaris [59]. In Sect. 5 we then show how we obtain the best heuristic quantum time complexity, by applying quantum search to the very recent HashSieve algorithm of Laarhoven [47], which in turn builds upon the NV-Sieve and the GaussSieve. Finally, in Sect. 8 we discuss quantum speed-ups for various other sieving algorithms, and in Sect. 9 we discuss why quantum search does not seem to lead to big asymptotic improvements in the time complexity of the Voronoi cell algorithm and enumeration algorithms.

## The provable ListSieve-Birthday algorithm of Pujol and Stehlé


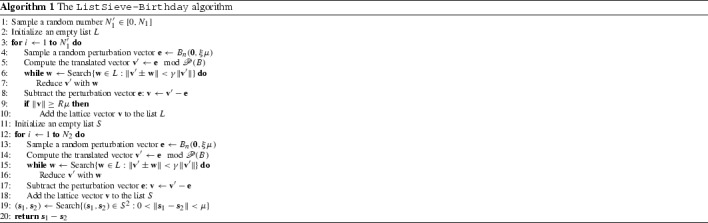


Using the birthday paradox [57], Pujol and Stehlé [65] showed that the constant in the exponent of the time complexity of the original ListSieve algorithm of Micciancio and Voulgaris [59], Sect. 3.1] can be reduced by almost $25\,\%$. The algorithm is presented in Algorithm 1. Here $\gamma = 1 - \frac{1}{n}$, $B_n(\mathbf {0}, \xi \mu )$ denotes the ball centered at $\mathbf {0}$ of radius $\xi \mu$, and the various other parameters will be discussed below.

### Description of the algorithm

The algorithm can roughly be divided in three stages, as follows.

First, the algorithm generates a long list $L$ of lattice vectors with norms between $R \mu$ and $\Vert B\Vert$. This ‘dummy’ list is used for technical reasons to make the proof strategy work. The number of samples used for generating this list is taken as a random variable, which again is done to make certain proof techniques work. Note that besides the actual lattice vectors $\mathbf {v}$, to generate this list we also consider slightly perturbed vectors $\mathbf {v}'$ which are not in the lattice, but are at most $\xi \mu$ away from $\mathbf {v}$. This is yet again a technical modification purely aimed at making the proofs work, as experiments show that without such perturbed vectors, these algorithms also work fine.

After generating $L$, we generate a fresh list of short lattice vectors $S$. The procedure for generating these vectors is similar to that of generating $T$, with two exceptions: (i) now all sampled lattice vectors are added to $S$ (regardless of their norms), and (ii) the vectors are reduced with the dummy list $L$ rather than with vectors in $S$. The latter guarantees that the vectors in $S$ are all independent and identically distributed.

Finally, when $S$ has been generated, we hope that it contains two distinct lattice vectors $\mathbf {s}_1$, $\mathbf {s}_2$ that are at most $\mu \approx \lambda _1(\fancyscript{L})$ apart. So we search $S \times S$ for a pair $(\mathbf {s}_1, \mathbf {s}_2)$ of close, distinct lattice vectors, and return their difference.

### Classical complexities

With a classical search applied to the subroutines in Lines 6, 15, and 19, Pujol and Stehlé analyzed that the costs of the algorithm are:

- Cost of generating $L$: $\tilde{O}(N_1 \cdot |L|) = 2^{(c_g + 2c_t)n + o(n)}$.
- Cost of generating $S$: $\tilde{O}(N_2 \cdot |L|) = 2^{(c_g + \frac{1}{2}c_b + c_t)n + o(n)}$.
- Cost of searching $S$ for a pair of close vectors: $\tilde{O}(|S|^2) = 2^{(2c_g + c_b)n + o(n)}$.
- Memory requirement of storing $S$ and $L$: $O(|S| + |L|) = 2^{\max (c_t, c_g + \frac{1}{2}c_b)n + o(n)}$.

The constants $c_b, c_t, c_g, N_1$ and $N_2$ above are defined as

1 $$\begin{aligned}&c_b = 0.401 + \log _2(R), \qquad \qquad \,\qquad \qquad \quad N_1 = 2^{(c_g + c_t)n + o(n)}, \end{aligned}$$

2 $$\begin{aligned}&c_t = 0.401 + \frac{1}{2}\log _2\left( 1 + \frac{2\xi }{R - 2\xi }\right) , \quad \qquad \quad N_2 = 2^{(c_g + c_b/2)n + o(n)} \end{aligned}$$

3 $$\begin{aligned}&c_g = \frac{1}{2}\log _2\left( \frac{4\xi ^2}{4\xi ^2 - 1}\right) . \end{aligned}$$

In [65] this led to the following result on the time and space complexities.

#### **Lemma 1**

[65] Let $\xi > \frac{1}{2}$ and $R > 2\xi$, and suppose $\mu > \lambda _1(\fancyscript{L})$. Then with probability at least $\frac{1}{16}$, the ListSieve-Birthday algorithm returns a lattice vector $\mathbf {s} \in \fancyscript{L} \setminus \{\mathbf {0}\}$ with $\Vert \mathbf {s}\Vert < \mu$, in time at most $2^{c_{{\text {time}}}n + o(n)}$ and space at most $2^{c_{{\text {space}}}n + o(n)}$, where $c_{{\text {time}}}$ and $c_{{\text {space}}}$ are given by

4 $$\begin{aligned} c_{{\text {time}}}= \max \left( c_g + 2c_t, c_g + \frac{c_b}{2} + c_t, 2c_g + c_b\right) , \quad c_{{\text {space}}}= \max \left( c_t, c_g + \frac{c_b}{2}\right) . \end{aligned}$$

By balancing $\xi$ and $R$ optimally, Pujol and Stehlé obtained the following result.

#### **Corollary 1**

[65] Letting $\xi \approx 0.9476$ and $R \approx 3.0169$, we obtain

5 $$\begin{aligned} c_{{\text {time}}}\approx 2.465, \quad c_{{\text {space}}}\approx 1.233. \end{aligned}$$

Thus, using polynomially many queries to the ListSieve-Birthday algorithm with these parameters, we can find a shortest vector in a lattice with probability exponentially close to $1$ using time at most $2^{2.465n + o(n)}$ and space at most $2^{1.233n + o(n)}$.

### Quantum complexities

Applying a quantum search subroutine to Lines 6, 15, and 19, we get the following costs for the quantum algorithm based on ListSieve-Birthday:

- Cost of generating $L$: $\tilde{O}(N_1 \cdot \sqrt{|L|}) = 2^{(c_g + \frac{3}{2}c_t)n + o(n)}$.
- Cost of generating $S$: $\tilde{O}(N_2 \cdot \sqrt{|L|}) = 2^{(c_g + \frac{1}{2}c_b + \frac{1}{2}c_t)n + o(n)}$.
- Cost of searching $S$ for a pair of close vectors: $\tilde{O}(\sqrt{|S|^2}) = 2^{(c_g + \frac{1}{2}c_b)n + o(n)}$.
- Memory requirement of storing $S$ and $L$: $O(|S| + |L|) = 2^{\max (c_t, c_g + \frac{1}{2}c_b)n + o(n)}$.

This leads to the following general lemma about the overall quantum time and space complexities.

#### **Lemma 2**

Let $\xi > \frac{1}{2}$ and $R > 2\xi$, and suppose $\mu > \lambda _1(\fancyscript{L})$. Then with probability at least $\frac{1}{16}$, the ListSieve-Birthday algorithm returns a lattice vector $\mathbf {s} \in \fancyscript{L} \setminus \{\mathbf {0}\}$ with $\Vert \mathbf {s}\Vert < \mu$ on a quantum computer in time at most $2^{q_{{\text {time}}}n + o(n)}$ and space at most $2^{q_{{\text {space}}}n + o(n)}$, where $q_{{\text {time}}}$ and $q_{{\text {space}}}$ are given by

6 $$\begin{aligned} q_{{\text {time}}}= \max \left( c_g + \frac{3c_t}{2}, c_g + \frac{c_b}{2} + \frac{c_t}{2}, c_g + \frac{c_b}{2}\right) , \quad q_{{\text {space}}}= \max \left( c_t, c_g + \frac{c_b}{2}\right) . \end{aligned}$$

Re-optimizing the parameters $\xi$ and $R$ subject to the given constraints, to minimize the overall time complexity, we obtain the following result.

#### **Theorem 1**

Letting $\xi \approx 0.9086$ and $R \approx 3.1376$, we obtain

7 $$\begin{aligned} q_{{\text {time}}}\approx 1.799, \quad q_{{\text {space}}}\approx 1.286. \end{aligned}$$

Thus, using polynomially many queries to the ListSieve-Birthday algorithm, we can find a shortest non-zero vector in a lattice on a quantum computer with probability exponentially close to $1$, in time at most $2^{1.799n + o(n)}$ and space at most $2^{1.286n + o(n)}$.

So the constant in the exponent of the time complexity decreases by about $27\ \%$ when using quantum search.

*Remark* If we generate $S$ in parallel, we can potentially achieve a time complexity of $2^{1.470n + o(n)}$, by setting $\xi \approx 1.0610$ and $R \approx 4.5166$. However, it would require exponentially many parallel quantum computers of size $O(n)$ to achieve a substantial theoretical speed-up over the $2^{1.799n + o(n)}$ of Theorem 1.

## The heuristic NV-Sieve algorithm of Nguyen and Vidick


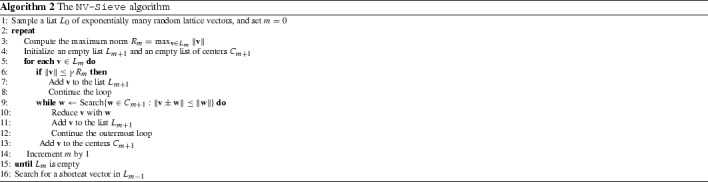


In 2008, Nguyen and Vidick [62] considered a heuristic, practical variant of the original AKS-Sieve algorithm of Ajtai et al. [3], which ‘provably’ returns a shortest vector under a certain natural, heuristic assumption. A slightly modified but essentially equivalent description of this algorithm is given in Algorithm 2.

### Description of the algorithm

The algorithm starts by generating a big list $L_0$ of random lattice vectors with length at most $n\Vert B\Vert$. Then, by repeatedly applying a sieve to this list, shorter lists of shorter vectors are obtained, until the list is completely depleted. In that case, we go back one step and search for the shortest vector in the last non-empty list.

The sieving step consists of splitting the previous list $L_m$ in a set of ‘centers’ $C_{m+1}$ and a new list of vectors $L_{m+1}$ that will be used for the next round. For each vector $\mathbf {v} \in L_m$, the algorithm first checks if a vector $\mathbf {w} \in C_m$ exists that is close to $\pm \mathbf {v}$. If this is the case, then we add the vector $\mathbf {v} \pm \mathbf {w}$ to $L_{m+1}$. Otherwise $\mathbf {v}$ is added to $C_{m+1}$. Since the set $C_{m+1}$ consists of vectors with a bounded norm and any two vectors in this list have a specified minimum pairwise distance, one can bound the size of $C_{m+1}$ from above using a result of Kabatiansky and Levenshtein [42] regarding sphere packings. In other words, $C_{m+1}$ will be sufficiently small, so that sufficiently many vectors are left for inclusion in the list $L_{m+1}$. After applying the sieve, we discard all vectors in $C_{m+1}$ and apply the sieve again to the vectors in $L_{m+1}$.

### Classical complexities

In Line 9 Algorithm 2, we have highlighted an application of a search subroutine that could be replaced by a quantum search. Using a standard classical search algorithm for this subroutine, under a certain heuristic assumption Nguyen and Vidick give the following estimate for the time and space complexity of their algorithm. Note that these estimates are based on the observation that the sizes of $S$ and $C$ are bounded from above by $2^{c_h n + o(n)}$, so that the total space complexity is at most $O(|S| + |C|) = 2^{c_h n + o(n)}$ and the total time complexity is at most $\tilde{O}(|S| \cdot |C|) = 2^{2c_h n + o(n)}$, assuming the sieve needs to be performed a polynomial number of times.

#### **Lemma 3**

[62] Let $\frac{2}{3} < \gamma < 1$ and let $c_h$ be defined as

8 $$\begin{aligned} c_h = -\log _2(\gamma ) - \frac{1}{2} \log _2\left( 1 - \frac{\gamma ^2}{4}\right) . \end{aligned}$$

Then the NV-Sieve algorithm heuristically returns a shortest non-zero lattice vector $\mathbf {s} \in \fancyscript{L} \setminus \{\mathbf {0}\}$ in time at most $2^{c_{{\text {time}}}n + o(n)}$ and space at most $2^{c_{{\text {space}}}n + o(n)}$, where $c_{{\text {time}}}$ and $c_{{\text {space}}}$ are given by

9 $$\begin{aligned} c_{{\text {time}}}= 2 c_h, \qquad c_{{\text {space}}}= c_h. \end{aligned}$$

To obtain a minimum time complexity, $\gamma$ should be chosen as close to $1$ as possible. Letting $\gamma \rightarrow 1$ Nguyen and Vidick thus obtain the following estimates for the complexity of their heuristic algorithm.

#### **Corollary 2**

[62] Letting $\gamma \rightarrow 1$, we obtain

10 $$\begin{aligned} c_{{\text {time}}}\approx 0.415, \quad c_{{\text {space}}}\approx 0.208. \end{aligned}$$

Thus, the NV-Sieve algorithm heuristically finds a shortest vector in time $2^{0.415n + o(n)}$ and space $2^{0.208n + o(n)}$.

### Quantum complexities

If we use a quantum search subroutine in Line 9, the complexity of this subroutine decreases from $\tilde{O}(|C|)$ to $\tilde{O}(\sqrt{|C|})$. Since this search is part of the bottleneck for the time complexity, applying a quantum search here will decrease the overall running time as well. Since replacing the classical search by a quantum search does not change the internal behavior of the algorithm, the estimates and heuristics are as valid as they were in the classical setting.

#### **Lemma 4**

Let $\frac{2}{3} < \gamma < 1$. Then the quantum version of the NV-Sieve algorithm heuristically returns a shortest non-zero lattice vector in time at most $2^{q_{{\text {time}}}n + o(n)}$ and space at most $2^{q_{{\text {space}}}n + o(n)}$, where $q_{{\text {time}}}$ and $q_{{\text {space}}}$ are given by

11 $$\begin{aligned} q_{{\text {time}}}= \frac{3}{2} \ c_h, \qquad q_{{\text {space}}}= c_h. \end{aligned}$$

Again, minimizing the asymptotic quantum time complexity corresponds to taking $\gamma$ as close to $1$ as possible, which leads to the following result.

#### **Theorem 2**

Letting $\gamma \rightarrow 1$, we obtain

12 $$\begin{aligned} q_{{\text {time}}}\approx 0.312, \quad q_{{\text {space}}}\approx 0.208. \end{aligned}$$

Thus, the quantum version of the NV-Sieve algorithm heuristically finds a shortest vector in time $2^{0.312n + o(n)}$ and space $2^{0.208n + o(n)}$.

In other words, applying quantum search to Nguyen and Vidick’s sieve algorithm leads to a $25\,\%$ decrease in the asymptotic exponent of the runtime.

## The heuristic GaussSieve algorithm of Micciancio and Voulgaris


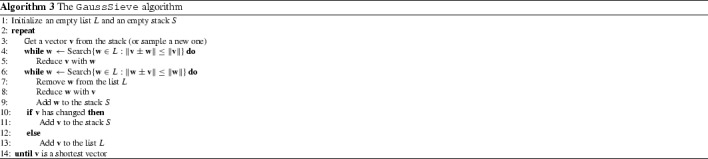


In 2010, Micciancio and Voulgaris [59] described a heuristic variant of their provable ListSieve algorithm, for which they could not give a (heuristic) bound on the time complexity, but which has a better heuristic bound on the space complexity, and has a better practical time complexity. The algorithm is described in Algorithm 3.

### Description of the algorithm

The algorithm is similar to the ListSieve-Birthday algorithm described earlier, with the following main differences: (i) we do not explicitly generate two lists $S$, $L$ to apply the birthday paradox in the proof; (ii) we do not use a geometric factor $\gamma < 1$ but always reduce a vector if it can be reduced; (iii) we also reduce existing list vectors $\mathbf {w} \in L$ with newly sampled vectors, so that each two vectors in the list are pairwise *Gauss-reduced*; and (iv) instead of specifying the number of iterations in advance, we run the algorithm until we get so many collisions that we are convinced we have found a shortest vector in our list.

### Classical complexities

Micciancio and Voulgaris state that the algorithm above has an experimental time complexity of about $2^{0.52n}$ and a space complexity which is most likely bounded by $2^{0.208n}$ due to the kissing constant [59], Sect. 5]. In practice this algorithm even seems to outperform the NV-Sieve algorithm of Nguyen and Vidick [62]. It is therefore sometimes conjectured that this algorithm also has a time complexity of the order $2^{0.415n + o(n)}$, and the apparent extra factor $2^{0.1n}$ in the experimental time complexity may come from non-negligible polynomial factors in low dimensions. Thus one might conjecture the following.

#### **Conjecture 1**

The GaussSieve algorithm heuristically returns a shortest non-zero lattice vector in time at most $2^{c_{{\text {time}}}n + o(n)}$ and space at most $2^{c_{{\text {space}}}n + o(n)}$, where $c_{{\text {time}}}$ and $c_{{\text {space}}}$ are given by

13 $$\begin{aligned} c_{{\text {time}}}\approx 0.415, \quad c_{{\text {space}}}\approx 0.208. \end{aligned}$$

Note that this algorithm is again (conjectured to be) quadratic in the space complexity, since each pair of list vectors needs to be compared and potentially reduced at least once (and at most a polynomial number of times) to make sure that the final list is Gauss-reduced.

### Quantum complexities

To this heuristic algorithm, we can again apply the quantum speed-up using quantum search. If the number of times a vector is compared with $L$ to look for reductions is polynomial in $n$, this then leads to the following result.

#### **Conjecture 2**

The quantum version of the GaussSieve algorithm heuristically returns a shortest non-zero lattice vector in time at most $2^{q_{{\text {time}}}n + o(n)}$ and space at most $2^{q_{{\text {space}}}n + o(n)}$, where $q_{{\text {time}}}$ and $q_{{\text {space}}}$ are given by

14 $$\begin{aligned} q_{{\text {time}}}\approx 0.312, \quad q_{{\text {space}}}\approx 0.208. \end{aligned}$$

This means that the exponent in the time complexity is again conjectured to be reduced by about $25\,\%$ using quantum search, and the exponents are the same as for the NV-Sieve algorithm. Since the GaussSieve seems to outperform the NV-Sieve in practice, applying quantum search to the GaussSieve will probably lead to better practical time complexities.

## The heuristic HashSieve algorithm of Laarhoven


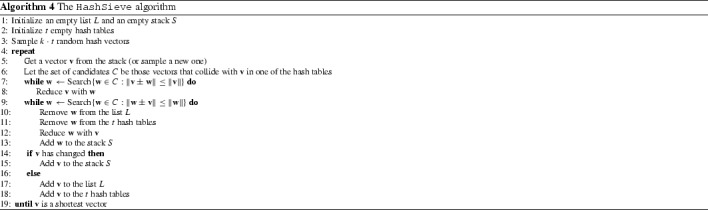


### Description of the algorithm

Recently, a modification of the GaussSieve and NV-Sieve algorithms was proposed in [47], improving the time complexity by using *angular locality-sensitive hashing* [20]. By storing low-dimensional sketches of the list vectors $\mathbf {w} \in L$ in these algorithms, it is possible to significantly reduce the number of list vectors $\mathbf {w}$ that need to be compared to a target vector $\mathbf {v}$ at the cost of increasing the space complexity. Using an exponential number of hash tables, where each list vector is assigned to one of the hash buckets in each hash table, and where vectors in the same bucket are more likely to be “close” in the Euclidean sense than vectors which are not in the same bin, we obtain the set of candidate close(st) vectors by computing which bucket this vector would have landed in, and taking all vectors from those bins as candidates.

### Classical complexities

With a proper balancing of the parameters, it can be guaranteed (heuristically) that the number of candidate vectors for each comparison is of the order $2^{0.1290n}$. This roughly corresponds to having $O(1)$ colliding vectors in each hash table, as the number of hash tables is also of the order $t = 2^{0.1290n}$, and this choice is optimal in the sense that this leads to a minimal time complexity of $2^{0.3366n}$; the space complexity is also $2^{0.3366n}$, and thus using even more hash tables increases the space complexity beyond the time complexity, thus also increasing the time complexity further. The exact choice of parameters is given below.

#### **Lemma 5**

[47], Cor. 1] Let $\log _2(t) \approx 0.129$. Then the HashSieve algorithm heuristically returns a shortest non-zero lattice vector in time at most $2^{c_{{\text {time}}}n + o(n)}$ and space at most $2^{c_{{\text {space}}}n + o(n)}$, where $c_{{\text {time}}}$ and $c_{{\text {space}}}$ are given by

15 $$\begin{aligned} c_{{\text {time}}}&\approx 0.337, \qquad c_{{\text {space}}}\approx 0.337. \end{aligned}$$

In other words, using $t \approx 2^{0.129 n}$ hash tables and a *hash length* of $k \approx 0.221n$, the time and space complexities of the HashSieve algorithm are balanced at $2^{0.337n + o(n)}$.

### Quantum complexities

With a quantum search on the set of candidates in Lines 7 and 9, we can further reduce the time complexity. The optimization changes in the sense that the time to search the list of candidates with quantum search is potentially reduced from $2^{(2 - \alpha ) c_n n + o(n)}$ to $2^{(\frac{3}{2} - \frac{1}{2} \alpha ) c_n n + o(n)}$, where $c_n = \log _2 N \approx 0.2075$ is the expected log-length of the list $L$ and $\alpha$ is defined in [47]. The numerical optimization of the parameters can be performed again, and leads to the following result.

#### **Theorem 3**

Let $\log _2(t) \approx 0.078$. Then the quantum HashSieve algorithm heuristically returns a shortest non-zero lattice vector in time at most $2^{q_{{\text {time}}}n + o(n)}$ and space at most $2^{q_{{\text {space}}}n + o(n)}$, where $q_{{\text {time}}}$ and $q_{{\text {space}}}$ are given by

16 $$\begin{aligned} q_{{\text {time}}}&\approx 0.286, \qquad q_{{\text {space}}}\approx 0.286. \end{aligned}$$

In other words, using $t \approx 2^{0.078n}$ hash tables and a hash length of $k \approx 0.134n$, the quantum time and space complexities of the algorithm are balanced at $2^{0.286n + o(n)}$.

It is possible to obtain a continuous trade-off between the quantum time and space complexities, by choosing $\log _2(t) \in [0, 0.07843]$ differently. Similar to Fig. 1 of [47], Fig. 1 shows the resulting trade-off, and a comparison with previous classical heuristic time complexities.

## The heuristic SphereSieve algorithm of Laarhoven and De Weger

### Description of the algorithm

Even more recently, another LSH-based modification of the NV-Sieve algorithm was proposed in [48], improving upon the time complexity of the HashSieve using *spherical* locality-sensitive hashing. This particular hash method, introduced by Andoni et al. [5, 6], works very well for data sets that (approximately) lie on the surface of a hypersphere, which is the case for the iterative sieving steps of the NV-Sieve. By dividing up the sphere into $2^{\varTheta (\sqrt{n})}$ regions in a way similar as in the 2-Level-Sieve of Wang et al. [79], it can be guaranteed that vectors have a significantly lower probability of ending up in the same region if their angle is large. Again using exponentially many hash tables, where each list vector is assigned to one of the hash buckets in each hash table, we obtain a set of candidate close(st) vectors by computing which bucket this vector would have landed in, and taking all vectors from those bins as candidates. The algorithm itself is a merge of the introduction of hash tables, as in the HashSieve, and the NV-Sieve of Nguyen and Vidick, with the important observation is that the hash functions used are different than in the HashSieve.

### Classical complexities

With a proper balancing of the parameters, it can be guaranteed (heuristically) that the number of candidate vectors for each comparison is of the order $2^{0.0896n}$, which is again similar to the number of hash tables, which is of the order $t = 2^{0.0896n}$. This choice is optimal in that this leads to a minimal time complexity of $2^{0.2972n}$; the space complexity is also $2^{0.2972n}$, and thus using even more hash tables increases the space complexity beyond the time complexity. The exact choice of parameters is given below.

#### **Lemma 6**

[47], Cor. 1] Let $\log _2(t) \approx 0.0896$. Then the SphereSieve algorithm heuristically returns a shortest non-zero lattice vector in time at most $2^{c_{{\text {time}}}n + o(n)}$ and space at most $2^{c_{{\text {space}}}n + o(n)}$, where $c_{{\text {time}}}$ and $c_{{\text {space}}}$ are given by

17 $$\begin{aligned} c_{{\text {time}}}&\approx 0.298, \qquad c_{{\text {space}}}\approx 0.298. \end{aligned}$$

In other words, using $t \approx 2^{0.0896 n}$ hash tables and a hash length of $k = \varTheta (\sqrt{n})$, the time and space complexities of the SphereSieve algorithm are balanced at $2^{0.298n + o(n)}$.

### Quantum complexities

With a quantum search on the set of candidates, we can again potentially reduce the time complexity. Again, the time to search the list of candidates with quantum search is reduced from $2^{(2 - \alpha ) c_n n + o(n)}$ to $2^{\big (\frac{3}{2} - \frac{1}{2} \alpha \big ) c_n n + o(n)}$, where $c_n = \log _2 N \approx 0.2075$ is the expected log-length of the list $L$ and bounds on $\alpha$ are defined in [48]. The numerical optimization of the parameters can be performed again, and leads to the following result.

#### **Theorem 4**

Let $\log _2(t) \approx 0.0413$. Then the quantum SphereSieve algorithm heuristically returns a shortest non-zero lattice vector in time at most $2^{q_{{\text {time}}}n + o(n)}$ and space at most $2^{q_{{\text {space}}}n + o(n)}$, where $q_{{\text {time}}}$ and $q_{{\text {space}}}$ are given by

18 $$\begin{aligned} q_{{\text {time}}}&\approx 0.268, \qquad q_{{\text {space}}}\approx 0.268. \end{aligned}$$

In other words, using $t \approx 2^{0.0413n}$ hash tables and a hash length of $k = \varTheta (\sqrt{n})$, the quantum time and space complexities of the algorithm are balanced at $2^{0.268n + o(n)}$.

Again, one may obtain a trade-off between the quantum time and space complexities, by choosing $\log _2(t) \in [0, 0.0413]$. This trade-off is shown in Fig. 1.

## The approximate ListSieve-Birthday analysis of Liu et al.

### Description of the algorithm

While most sieving algorithms are concerned with finding exact solutions to the shortest vector problem (i.e., finding a lattice vector whose norm is the minimum over all non-zero lattice vectors), in many cryptographic applications, finding a short (rather than shortest) vector in the lattice also suffices. Understanding the costs of finding approximations to shortest vectors (i.e., solving SVP$_{\delta }$ with $\delta > 1$) may therefore be as important the costs of exact SVP.

In 2011, Liu et al. [54] analyzed the impact of this relaxation of SVP on lattice sieving algorithms. In particular, they analyzed the ListSieve-Birthday algorithm of Sect. 2, taking into account the fact that an approximate solution is sufficient. The algorithm, described in [54], Algorithm 1], is effectively identical to the original ListSieve-Birthday algorithm of Pujol and Stehlé [65].

### Classical complexities

Intuitively, the effect of large $\delta$ can be understood as that the impact of the use of perturbed lattice vectors (rather than actual lattice vectors) becomes less and less. In the limit of large $\delta$, the impact of perturbations disappears (although it still guarantees correctness of the algorithm), and we get the same upper bound on the list size of $2^{0.401n}$ as obtained for the perturbation-free heuristic version of the *ListSieve*, the GaussSieve [59]. Since the runtime of the sieve remains quadratic in the list size, this leads to a time complexity of $2^{0.802n}$.

#### **Lemma 7**

[54], Lemma 10] The ListSieve-Birthday algorithm heuristically returns a solution to SVP$_{\delta }$ in time at most $2^{c_{{\text {time}}}n + o(n)}$ and space at most $2^{c_{{\text {space}}}n + o(n)}$, where $c_{{\text {time}}}$ and $c_{{\text {space}}}$ are given by

19 $$\begin{aligned} c_{{\text {time}}}&\approx 0.802, \qquad c_{{\text {space}}}\approx 0.401. \end{aligned}$$

### Quantum complexities

As expected, using quantum search in the ListSieve-Birthday algorithm leads to a gain in the exponent of $25\,\%$; a single search can be done in time $\tilde{O}(\sqrt{N})$, leading to a total time complexity of $\tilde{O}(N^{3/2})$ rather than $\tilde{O}(N^2)$.

#### **Theorem 5**

The quantum ListSieve-Birthday algorithm heuristically returns a $\delta$-approximation to the shortest non-zero lattice vector in time at most $2^{q_{{\text {time}}}n + o(n)}$ and space at most $2^{q_{{\text {space}}}n + o(n)}$, where $q_{{\text {time}}}$ and $q_{{\text {space}}}$ are given by

20 $$\begin{aligned} q_{{\text {time}}}&\approx 0.602, \qquad q_{{\text {space}}}\approx 0.401. \end{aligned}$$

## Other sieve algorithms

### The provable AKS-Sieve of Ajtai et al.

Ajtai et al. [3] did not provide an analysis with concrete constants in the exponent in their original paper of the AKS-Sieve. We expect that it is possible to speed up this version of the algorithm using quantum search as well, but instead we consider several subsequent variants that are easier to analyse.

The first of these was by Regev [67], who simplified the presentation and gave concrete constants for the running time and space complexity. His variant is quadratic in the list size, which is bounded by $2^{8n+o(n)}$, leading to a worst-case time complexity of $2^{16n+o(n)}$. Using quantum search, the exponent in the runtime decreases by $25\,\%$, which results in a run-time complexity of $2^{12n+o(n)}$.

Nguyen and Vidick [62] improved this analysis by carefully choosing the parameters of the algorithm, which resulted in a space complexity of $2^{2.95n+o(n)}$. The running time of $2^{5.9n+o(n)}$ is again quadratic in the list size, and can be improved using quantum search by $25\,\%$ to $2^{4.425n}$.

Micciancio and Voulgaris improve the constant as follows. Say that the initial list contains $2^{c_0n+o(n)}$ vectors, the probability that a point is not a collision at the end is $p = 2^{-c_un +o(n)}$ and the maximum number of points used as centers is $2^{c_sn+o(n)}$. Each step of sieving costs $2^{(c_0+c_s)n+o(n)}$ time. Now, after $k$ sieving steps of the algorithm the number of points will be $|P_k| = \tilde{O}(2^{c_0n} - k2^{c_sn})$, which results in $|V_k| = \tilde{O}((2^{c_0n} - k2^{c_sn})/2^{c_un})\approx 2^{c_Rn+o(n)}$ distinct non-perturbed lattice points. This set $P_k$ is then searched for a pair of lattice vectors such that the difference is a non-zero shortest vector, which classically costs $|V_k|\cdot |P_k| = 2^{(2c_R+c_u)n+o(n)}$.

*Classical complexities* In the above description, we have the following correspondence:

21 $$\begin{aligned} c_u&= \log \left( \frac{\xi }{\sqrt{\xi ^2-0.25}}\right) ,&\qquad \quad c_s&= 0.401 + \log \left( \frac{1}{\gamma }\right) , \end{aligned}$$

22 $$\begin{aligned} c_R&= 0.401 + \log \left( \xi \left( 1+\frac{1}{1-\gamma }\right) \right) ,&\qquad \quad c_0&= \max \{c_s,c_R+c_u\}. \end{aligned}$$

where $\xi \in [0.5,\frac{1}{2}\sqrt{2})$ and $\gamma < 1$. The space complexity is $2^{c_0n+o(n)}$ and the time complexity is $2^{c_Tn+o(n)}$ with

23 $$\begin{aligned} c_{{\text {time}}}= \max \{c_0+c_s,\ 2c_R+c_u\}, \qquad c_{{\text {space}}}= c_0. \end{aligned}$$

Optimizing $\xi$ and $\gamma$ to minimize the classical time complexity leads to $\xi \approx 0.676$ and $\gamma \approx 0.496$ which gives space $2^{1.985n+o(n)}$ and time $2^{3.398n+o(n)}$.

*Quantum complexities* Quantum searching in the sieving step speeds up this part of the algorithm to $2^{\big (c_0+\frac{1}{2}c_s\big )n+o(n)}$. In the final step quantum search can be used to speed up the search to $\sqrt{|V_k|}\cdot |P_k| = \sqrt{2^{c_Rn}}\cdot 2^{(c_R+c_u)n+o(n)} = 2^{\big (\frac{3}{2}c_R+c_u\big )n+o(n)}$. Thus, the exponents of the quantum time and space become

24 $$\begin{aligned} q_{{\text {time}}}= \max \left\{ c_0+\frac{1}{2}\ c_s, \ \frac{3}{2} \ c_R + c_u\right\} , \qquad q_{{\text {space}}}= c_0. \end{aligned}$$

Optimizing gives $\xi \rightarrow \frac{1}{2}\sqrt{2}$ and $\gamma = 0.438$, which results in a space complexity of $2^{1.876n+o(n)}$ and running time of $2^{2.672n+o(n)}$.

### The provable ListSieve of Micciancio and Voulgaris

The provable ListSieve algorithm of Micciancio and Voulgaris [59] was introduced as a provable variant of their heuristic GaussSieve algorithm, achieving a better time complexity than with the optimized analysis of the AKS-Sieve. Instead of starting with a big list and repeatedly applying a sieve to reduce the length of the list (and the norms of the vectors in the list), the ListSieve builds a longer and longer list of vectors, where each new vector to be added to the list is first reduced with all other vectors in the list. (But unlike the GaussSieve, vectors already in the list are never modified.) Complete details of the algorithm and its analysis can be found in [59].

*Classical complexities* First, for $\xi \in (0.5, 0.7)$ we write

25 $$\begin{aligned} c_1 = 0.401 + \log _2\left( \xi + \sqrt{1 + \xi ^2}\right) , \qquad c_2 = \log _2\left( \frac{\xi }{\sqrt{\xi ^2 - \frac{1}{4}}}\right) . \end{aligned}$$

Then the ListSieve algorithm has a provable complexity of at most $2^{(2c_1 + c_2)n + o(n)}$ (time) and $2^{c_1 n + o(n)}$ (space) for any $\xi$ in this interval. Minimizing the time complexity leads to $\xi \approx 0.685$, with a time complexity of $2^{3.199n + o(n)}$ and a space complexity of $2^{1.325n + o(n)}$.

*Quantum complexities* Using quantum search, it can be seen that the inner search of the list of length $N = 2^{c_1 n + o(n)}$ can now be performed in time $2^{\frac{1}{2} c_1 n + o(n)}$. Thus the total time complexity becomes $2^{\big (\frac{3}{2} c_1 + c_2\big )n + o(n)}$ now. Optimizing for $\xi$ shows that the optimum is at the boundary of $\xi \rightarrow 0.7$.

Looking a bit more closely at Micciancio and Voulgaris’ analysis, we see that the condition $\xi < 0.7$ comes from the condition that $\xi \times \mu \le \frac{1}{2} \lambda _1^2$. Taking $\mu < 1.01 \lambda _1$ then approximately leads to the given bound for $\xi$, and since in the classical case the optimum does not lie at the boundary anyway, this was sufficient for Micciancio and Voulgaris. However, now that the optimum is at the boundary, we can see that we can slightly push the boundary further and slightly relax the condition $\xi < 0.7$. For any constant $\varepsilon > 0$ we can also let $\mu < (1 + \epsilon ) \lambda _1$ without losing any performance of the algorithm, and for small $\varepsilon$ this roughly translates to the bound $\xi < \frac{1}{2} \sqrt{2} \approx 0.707$.

With this adjustment in their analysis, the optimum is at the boundary of $\xi \rightarrow \frac{1}{2} \sqrt{2}$, in which case we get a quantum time complexity of $2^{ 2.527n + o(n)}$ and a space complexity of $2^{ 1.351n + o(n)}$.

### The provable AKS-Sieve-Birthday algorithm of Hanrot et al.

Hanrot, Pujol and Stehlé [38] described a speed-up for the AKS-Sieve using the birthday paradox [57], similar to the speed-up that Pujol and Stehlé describe for Listsieve. Recall that the AKS-Sieve consists of an initial sieving step that generates a list of reasonably small vectors, followed by a pairwise comparison of the remaining vectors because the difference of at least one pair is expected to be a shortest non-zero vector. If this list of reasonably small vectors are independent and identically distributed, the number of vectors required is reduced to the square root of the original amount by the birthday paradox. They describe how this can be done for the AKS-Sieve, which requires fixing what vectors are used as centers for every iteration. This means that when a perturbed vector does not lie close to any of the fixed centers in the generation of the list of small vectors, it is discarded.

*Classical complexities* For $\xi > \frac{1}{2}$ and $\gamma < 1$ we write

26 $$\begin{aligned} c_t&= 0.401 - \log _2(\gamma ), c_b = 0.401 + \log _2\left( \xi + \frac{\xi }{1 - \gamma }\right) , c_g = -\frac{1}{2}\log _2\left( 1 - \frac{1}{4\xi ^2}\right) . \end{aligned}$$

The AKS-Sieve-Birthday algorithm now has a time complexity of $2^{c_\mathrm {time}n + o(n)}$ and a space complexity of $2^{c_\mathrm {space}n+o(n)}$, where

27 $$\begin{aligned} c_{{\text {time}}}= c_g+\max \left\{ 2c_t, c_g + c_t + \frac{c_b}{2}, c_g + c_b\right\} , \quad c_{{\text {space}}}= c_g + \max \left\{ c_t, \frac{c_b}{2}\right\} . \end{aligned}$$

Working out the details, the classically optimized constants are $\xi \rightarrow 1$ and $\gamma \approx 0.609$ leading to a time complexity of $2^{2.64791n + o(n)}$ and a space complexity of $2^{1.32396n + o(n)}$.

*Quantum complexities* Replacing various steps with quantum search gives the same space exponent $q_{{\text {space}}}= c_{{\text {space}}}$ as in the classical case, and leads to the following time exponent:

28 $$\begin{aligned} q_{{\text {time}}}= c_g + \max \left\{ \frac{3 c_t}{2}, \frac{c_t}{2} + \frac{c_b}{2}, \frac{c_g}{2} + c_t + \frac{c_b}{4}, \frac{c_g}{2} + \frac{3c_b}{4}\right\} . \end{aligned}$$

Optimizing the parameters to obtain the lowest quantum time complexity, we get the same constants $\xi \rightarrow 1$ and $\gamma \approx 0.609$ leading to a time complexity of $2^{1.98548n + o(n)}$ (which is exactly a $25\,\%$ gain in the exponent) and a space complexity of $2^{1.32366n + o(n)}$.

### The heuristic 2-Level-Sieve of Wang et al.

To improve upon the time complexity of the algorithm of Nguyen and Vidick, Wang et al. [79] introduced a further trade-off between the time complexity and the space complexity. Their algorithm uses two lists of centers $C_1$ and $C_2$ and two geometric factors $\gamma _1$ and $\gamma _2$, instead of the single list $C$ and single geometric factor $\gamma$ in the algorithm of Nguyen and Vidick. For details, see [79].

*Classical complexities* The classical time complexity of this algorithm is bounded from above by $\tilde{O}(|C_1| \cdot |C_2| \cdot (|C_1| + |C_2|))$, while the space required is at most $O(|C_1| \cdot |C_2|)$. Optimizing the constants $\gamma _1$ and $\gamma _2$ in their paper leads to $(\gamma _1, \gamma _2) \approx (1.0927, 1)$, with an asymptotic time complexity of less than $2^{0.384n + o(n)}$ and a space complexity of about $2^{0.256n + o(n)}$.

*Quantum complexities* By using the quantum search algorithm for searching the lists $C_1$ and $C_2$, the time complexity is reduced to $\tilde{O}(|C_1| \cdot |C_2| \cdot \sqrt{|C_1| + |C_2|})$, while the space complexity remains $O(|C_1| \cdot |C_2|)$. Re-optimizing the constants for a minimum quantum time complexity leads to $(\gamma _1, \gamma _2) \approx (\sqrt{2}, 1)$, leading to the same time and space complexities as the quantum-version of the algorithm of Nguyen and Vidick. Due to the simpler algorithm and smaller constants, a quantum version of the algorithm of Nguyen and Vidick will most likely be more efficient than a quantum version of the algorithm of Wang et al.

### The heuristic 3-Level-Sieve of Zhang et al.

To further improve upon the time complexity of the 1-Level-Sieve (NV-Sieve) of Nguyen and Vidick and the 2-Level-Sieve of Wang et al. [79], Zhang et al. [80] introduced the 3-Level-Sieve, with a further trade-off between the time complexity and the space complexity. Their algorithm generalizes the 2-Level-Sieve with two lists of centers (with different radii) to three lists of centers. For the complete details of this algorithm, see [80].

*Classical complexities* The classical time complexity of this algorithm is bounded by $\tilde{O}(|C_1| \cdot |C_2| \cdot |C_3| \cdot (|C_1| + |C_2| + |C_3|))$, while the space required is at most $O(|C_1| \cdot |C_2| \cdot |C_3|)$. Optimizing the constants $\gamma _1, \gamma _2, \gamma _3$ leads to $(\gamma _1, \gamma _2, \gamma _3) \approx (1.1399, 1.0677, 1)$, with an asymptotic time complexity of less than $2^{0.378n + o(n)}$ and a space complexity of about $2^{0.283n + o(n)}$.

*Quantum complexities* By using the quantum search algorithm for searching the lists $C_{1,2,3}$, the time complexity is reduced to $\tilde{O}(|C_1| \cdot |C_2| \cdot |C_3| \cdot \sqrt{|C_1| + |C_2| + |C_3|})$, while the space complexity remains $O(|C_1| \cdot |C_2| \cdot |C_3|)$. Re-optimizing the constants for a minimum time complexity leads to $(\gamma _1, \gamma _2, \gamma _3) \approx (\sqrt{2}, \sqrt{2}, 1)$, again leading to the same time and space complexities as the quantum-version of the algorithm of Nguyen and Vidick and the quantum version of the 2-Level-Sieve of Wang et al. Again the hidden polynomial factors of the 3-Level-Sieve are much larger, so the quantum version of the NV-Sieve of Nguyen and Vidick is most likely faster.

### The heuristic Overlattice-Sieve of Becker et al.

The Overlattice-Sieve works by decomposing the lattice into a sequence of overlattices such that the lattice at the bottom corresponds to the challenge lattice, whereas the lattice at the top corresponds to a lattice where enumerating short vectors is easy due to orthogonality. The algorithm begins by enumerating many short vectors in the top lattice and then iteratively moves down through the sequence of lattices by combining short vectors in the overlattice to form vectors in the lattice directly below it in the sequence. It keeps only the short non-zero vectors that are formed in this manner and uses them for the next iteration. In the last iteration, it generates short vectors in the challenge lattice, and these give a solution to the shortest vector problem. Since the algorithm actually works on cosets of the lattice, it is more naturally seen as an algorithm for the closest vector problem, which it solves as well. The algorithm relies on the assumption that the Gaussian Heuristic holds in all the lattices in the sequence.

More specifically, at any one time the algorithm deals with $\beta ^n$ vectors that are divided into $\alpha ^n$ buckets with on average $\beta ^n/\alpha ^n$ vectors per bucket. These buckets are divided into pairs such that any vector from a bucket and any vector from its paired bucket combine into a lattice vector in the sublattice. Therefore, exactly $\beta ^{2n}/\alpha ^{n}$ combinations need to be made in each iteration.

*Classical complexities* The above leads to a classical running time of $\tilde{O}(\beta ^{2n}/\alpha ^{n})$ and a space complexity of $\tilde{O}(\beta ^n)$, under the constraints that

$$\begin{aligned} 1 < \alpha < \sqrt{2}, \quad \alpha ^n \in \mathbb {Z}, \quad \beta \sqrt{1 - \frac{\alpha ^2}{4}} \ge 1 + \varepsilon _n, \end{aligned}$$

where $\varepsilon _n$ is some function that decreases towards $0$ as $n$ grows. Optimizing $\alpha$ and $\beta$ for the best time complexity gives $\alpha = \sqrt{\frac{4}{3}}$ and $\beta = \sqrt{\frac{3}{2}}$ for a running time of $2^{0.3774n+o(n)}$ and a space complexity of $2^{0.2925n+o(n)}$.

*Quantum complexities* By using the quantum search algorithm to search for suitable combinations for every vector, the running time can be reduced to $\tilde{O}(\beta ^{3n/2}/\alpha ^{n/2})$. This is optimal for $\alpha \rightarrow 1$, $\beta = \sqrt{\frac{4}{3}}$, which gives a quantum time complexity of $2^{0.311n + o(n)}$ and a space complexity of $2^{0.2075n + o(n)}$. Interestingly, in the classical case there is a trade-off between $\alpha$ and $\beta$, which allows for a bigger $\alpha$ (reducing the running time) at the cost of increasing $\beta$ (increasing the space complexity). In the quantum case, this trade-off is no longer possible: increasing $\alpha$ and $\beta$ actually leads to a larger running time as well as a larger space complexity. Thus, the resulting quantum complexity is heuristically no better than the other sieving algorithms, but this algorithm solves CVP as well as SVP.

## Other SVP algorithms

### Enumeration algorithms

In enumeration, all lattice vectors are considered inside a giant ball around the origin that is known to contain at least one lattice vector. Let $\fancyscript{L}$ be a lattice with basis $\{\mathbf {b}_1, \dots , \mathbf {b}_n\}$. Consider each lattice vector $\mathbf {u} \in \fancyscript{L}$ as a linear combination of the basis vectors, i.e., $\mathbf {u} = \sum _i u_i \mathbf {b}_i$. Now, we can represent each lattice vector by its coefficient vector $(u_1,\dots , u_n)$. We would like to have all combinations of values for $(u_1,\dots , u_n)$ such that the corresponding vector $\mathbf {u}$ lies inside the ball. We could try any combination and see if it lies within the ball by computing the norm of the corresponding vector, but there is a smarter way that ensures we only consider vectors that lie within the ball and none that lie outside.

To this end, enumeration algorithms search from right to left, by identifying all values for $u_n$ such that there might exist $u_1', \dots , u_{n-1}'$ such that the vector corresponding to $(u_1', \dots , u_{n-1}',u_n)$ lies in the ball. To identify these values $u_1', \dots , u_{n-1}'$, enumeration algorithms use the Gram–Schmidt orthogonalization of the lattice basis as well as the projection of lattice vectors. Then, for each of these possible values for $u_n$, the enumeration algorithm considers all possible values for $u_{n-1}$ and repeats the process until it reaches possible values for $u_1$. This leads to a search which is serial in nature, as each value of $u_n$ will lead to different possible values for $u_{n-1}$ and so forth. Unfortunately, we can only really apply the quantum search algorithm to problems where the list of objects to be searched is known in advance.

One might suggest to forego the smart way to find short vectors and just search all combinations of $(u_1, \dots , u_n)$ with appropriate upper and lower bounds on the different $u_i$’s. Then it becomes possible to apply quantum search, since we now have a predetermined list of vectors and just need to compute the norm of each vector. However, it is doubtful that this will result in a faster algorithm, because the recent heuristic changes by Gama et al. [31] have reduced the running time of enumeration dramatically (roughly by a factor $2^{n/2}$) and these changes only complicate the search area further by changing the ball to an ellipsoid. There seems to be no simple way to apply quantum search to the enumeration algorithms that are currently used in practice, but perhaps the algorithms can be modified in some way.

### The Voronoi cell algorithm

Consider a set of points in the Euclidean space. For any given point in this set, its Voronoi cell is defined as the region that contains all vectors that lie closer to this point than to any of the other points in the set. Now, given a Voronoi cell, we define a relevant vector to be any vector in the set whose removal from the set will change this particular Voronoi cell. If we pick our lattice as the set and we consider the Voronoi cell around the zero vector, then any shortest vector is also a relevant vector. Furthermore, given the relevant vectors of the Voronoi cell we can solve the closest vector problem in $2^{2n + o(n)}$ time.

So how can we compute the relevant vectors of the Voronoi cell of a lattice $\fancyscript{L}$? Micciancio and Voulgaris [58] show that this can be done by solving $2^n - 1$ instances of CVP in the lattice $2 \fancyscript{L}$. However, in order to solve CVP we would need the relevant vectors which means we are back to our original problem. Micciancio and Voulgaris show that these instances of CVP can also be solved by solving several related CVP instances in a lattice of lower rank. They give a basic and an optimized version of the algorithm. The basic version only uses LLL as preprocessing and solves all these related CVP instances in the lower rank lattice separately. As a consequence, the basic algorithm runs in time $2^{3.5n + o(n)}$ and in space $2^{n + o(n)}$. The optimized algorithm uses a stronger preprocessing for the lattice basis, which takes exponential time. But since the most expensive part is the computation of the Voronoi relevant vectors, this extra preprocessing time does not increase the asymptotic running time as it is executed only once. In fact, having the reduced basis decreases the asymptotic running time to $\tilde{O}(2^{3n})$. Furthermore, the optimized algorithm employs a trick that allows it to reduce $2^k$ CVP instances in a lattice of rank $k$ to a single instance of an enumeration problem related to the same lattice. The optimized algorithm solves CVP in time $\tilde{O}(2^{2n})$ using $\tilde{O}(2^n)$ space.

Now, in the basic algorithm, it would be possible to speed up the routine that solves CVP given the Voronoi relevant vectors using a quantum computer. It would also be possible to speed up the routine that removes non-relevant vectors from the list of relevant vectors using a quantum computer. Combining these two changes gives a quantum algorithm with an asymptotic running time $\tilde{O}(2^{2.5n})$, which is still slower than the optimized classical algorithm. It is not possible to apply these same speedups to the optimized algorithm due to the aforementioned trick with the enumeration problem. The algorithm to solve this enumeration problem makes use of a priority queue, which means the search is not trivially parallellized. Once again, there does not seem to be a simple way to apply quantum search to this special enumeration algorithm. However, it may be possible that the algorithm can be modified in such a way that quantum search can be applied.

### Discrete Gaussian sampling

A very recent method for finding shortest vectors in lattices is based on sampling and combining lattice vectors sampled from a discrete Gaussian on the lattice. Given a lattice, a discrete Gaussian distribution on the lattice is what you might expect it to be; the probability of sampling a non-lattice vector is $0$, and the probability of sampling a lattice vector $\mathbf {x}$ is proportional to $\exp -O(\Vert \mathbf {x}\Vert ^2)$, comparable to a regular multivariate Gaussian distribution. These discrete Gaussians are commonly used in lattice-based cryptographic primitives, such as lattice-based signatures [26, 56], and it is folklore that sampling from a discrete Gaussian distribution with a very large standard deviation is easy, while sampling from a distribution with a small standard deviation (often sampling short vectors) is hard.

The idea of Aggarwal et al. [1] to solve SVP with discrete Gaussian sampling is as follows. First, many vectors are sampled from a discrete Gaussian with large standard deviation. Then, to find shorter and shorter lattice vectors, list vectors are combined and averaged to obtain samples from a Gaussian with a smaller standard deviation. More precisely, two samples from a discrete Gaussian with width $\sigma$ can be combined and averaged to obtain a sample from a discrete Gaussian with width $\sigma /\sqrt{2}$. To be able to combine and average list vectors, they need to be in the same coset of $2\fancyscript{L}$, which means that $2^n$ buckets are stored, and within each bucket (coset) vectors are combined to obtain new, shorter samples. Overall, this leads to a time complexity of $2^{n + o(n)}$ for SVP, also using space $2^{n + o(n)}$.

The ideas of this algorithm are actually quite similar to the Overlattice-Sieve of Becker et al. [9] which we discussed in Sect. 8.6. Vectors are already stored in buckets, and so searching for vectors in the same coset is not costly at all. In this algorithm, the number of vectors in each bucket is even sub-exponential in $n$, so a quantum search speed-up does not seem to bring down the asymptotic time or space complexities at all. Due to the number of cosets of $2\fancyscript{L}$ ($2^n$), this algorithm seems (classically and quantumly) bound by a time and space complexity of $2^{n + o(n)}$, which it already achieves.

## Footnotes

1 At the time of the initial submission of this paper, the result of Micciancio and Voulgaris was the best provable asymptotic result for classical SVP (and CVP) solvers to date. The paper [1], provably solving SVP in time $2^{n + o(n)}$, appeared only in December 2014.

2 Here $o_{\delta }(n)$ corresponds to a function $f(\delta , n)$ satisfying $\lim \limits _{\delta \rightarrow \infty } \lim \limits _{n \rightarrow \infty } \frac{1}{n} f(\delta , n) \rightarrow 0$.
